# Global antibiotic use during the COVID-19 pandemic: analysis of pharmaceutical sales data from 71 countries, 2020–2022

**DOI:** 10.1016/j.eclinm.2023.101848

**Published:** 2023-02-06

**Authors:** Arindam Nandi, Simone Pecetta, David E. Bloom

**Affiliations:** a The Population Council, New York, USA; b One Health Trust, Washington DC, USA; c Research and Development Center, GlaxoSmithKline, Siena, Italy; d Department of Global Health and Population, Harvard T.H. Chan School of Public Health, Boston, MA, USA

**Keywords:** COVID-19, Antibiotic use, AMR, Antimicrobial resistance

## Abstract

**Background:**

Despite bacterial coinfection rates of less than 10%, antibiotics are prescribed to an estimated 75% of patients with COVID-19, potentially exacerbating antimicrobial resistance. We estimated the associations of COVID-19 cases and vaccinations with global antibiotic sales during the first two years of the COVID-19 pandemic.

**Methods:**

We obtained monthly data on broad-spectrum antibiotic sales volumes (cephalosporins, penicillins, macrolides, and tetracyclines) in 71 countries during March 2020–May 2022 from the IQVIA MIDAS® database. These data were combined with country-month-level COVID-19 case and vaccination data from Our World in Data. We used least squares (pooled) and fixed-effects panel data regression models, accounting for country characteristics, to estimate the associations between antibiotic sales volumes and COVID-19 cases and vaccinations per 1000 people.

**Findings:**

Sales of all four antibiotics fell sharply during April and May 2020, followed by a gradual rise to near pre-pandemic levels through May 2022. In fixed-effects regression models, a 10% increase in monthly COVID-19 cases was associated with 0.2%–0.3% higher sales of cephalosporins, 0.2%–0.3% higher sales of penicillins, 0.4%–0.6% higher sales of macrolides, and 0.3% higher sales of all four antibiotics combined per 1000 people. Across continents, a 10% increase in monthly COVID-19 cases was associated with 0.8%, 1.3%, and 1.5% higher macrolides sales in Europe, North America, and Africa respectively. Sales of other antibiotics across continent were also positively associated with COVID-19 cases, although the estimated associations were smaller in magnitude. No consistent associations were observed between antibiotic sales and COVID-19 vaccinations. Results from pooled regression analysis were similar to those from the fixed-effects models.

**Interpretation:**

Antibiotic sales were positively associated with COVID-19 cases globally during 2020–2022. Our findings underline that antibiotic stewardship in the context of COVID-19 remains essential.

**Funding:**

10.13039/100000865Bill & Melinda Gates Foundation.

**Research in context**

**Evidence before this study**

We searched PubMed and Google Scholar databases on October 15, 2022, using search terms that included combinations of the phrases “SARS-CoV-2,” “COVID-19,” “COVID,” “coronavirus pandemic,” “antibiotic use,” “antimicrobial resistance,” and “AMR,” and considered articles in English without any date restriction. We identified several studies of antibiotic use in patients with COVID-19 in clinical settings, including one meta-analysis, and four studies of overall (including non-COVID) antibiotic use in the United States, Canada, and India in 2020.

**Added value of this study**

This study is the first to estimate the associations of global and regional antibiotic use with COVID-19 cases and vaccinations during 2020–2022. Our findings show that monthly sales of cephalosporins, penicillins, and macrolides were associated positively with COVID-19 cases. No associations were evident between antibiotic sales and COVID-19 vaccinations.

**Implications of all the available evidence**

Guidelines preventing unnecessary antibiotic use in patients with COVID-19 must be developed and implemented globally to prevent rise in antimicrobial resistance.

## Introduction

Antimicrobial resistance (AMR) is an urgent global health threat. An estimated 5 million deaths worldwide were associated with AMR in 2019, of which 1.3 million deaths were directly attributable to AMR.^1^ The cumulative global economic burden of AMR by 2050 is estimated to be $100–$210 trillion or approximately 1% of global gross domestic product.^2^

Antibiotic use, especially overuse or inappropriate use including for viral infections such as the flu and common cold, is a major driver of AMR globally.^3^, ^4^, ^5^, ^6^, ^7^, ^8^ Global antibiotic consumption increased by 65% in 2000–2015, mainly due to high consumption levels in high-income countries (HICs).^9^ However, per capita consumption in low- and middle-income countries (LMICs) is growing rapidly due to improving standards of living and affordability of care and convergence with HICs.^9^ Furthermore, global per capita consumption of antibiotics from the World Health Organization's (WHO's) Watch list—those that should be used less frequently than WHO Access antibiotics (widely accessible first- and second-line treatment options) due to their higher AMR potential—increased by 91% during 2000–2015.^10^ Per capita consumption of Watch antibiotics increased by 165% in LMICs during this period, as compared with a 28% rise in HICs.^10^

The COVID-19 pandemic renewed attention to the need to tackle antibiotic misuse and AMR. Although less than 10% of hospitalised and community-based patients with COVID-19 worldwide are diagnosed with a secondary bacterial infection requiring antibiotics, an estimated 75% of patients receive antibiotic prescriptions.^11^, ^12^, ^13^, ^14^, ^15^, ^16^ The U.S. Centers for Disease Control and Prevention estimates that antibiotic resistance in the United States increased 15% during 2019–2020, leading to 29,400 additional deaths, of which 40% were from hospital-acquired infections.^17^

While clinical studies of COVID-19 case management show heightened antibiotic use, the full effect of the pandemic on global antibiotic consumption, including non-COVID-19 use, has yet to be studied. Nonpharmaceutical interventions (NPIs) for controlling COVID-19 spread such as lockdowns, travel restrictions, and use of masks may have reduced the transmission of many non-COVID infectious diseases, such as the seasonal flu, that are incorrectly treated with antibiotics.^18^, ^19^, ^20^, ^21^ Health system disruptions may have reduced access to and use of antibiotics for non-COVID illnesses.^22^^,^^23^ However, private and off-label use—including prophylactic use—of antibiotics during COVID-19 surges has been documented in some LMICs,^24^^,^^25^ and return to normalcy in 2021 and 2022 may have been associated with higher prevalence of non-COVID infections and related antibiotic use globally.^26^, ^27^, ^28^ Finally, routine childhood immunization coverage decreased globally due to health system constraints, which might have increased the prevalence of vaccine-preventable diseases and associated antibiotic use.^29^, ^30^, ^31^, ^32^ Understanding the systemic effect of the pandemic on overall antibiotic use given these counteracting factors is important for developing antimicrobial stewardship programs for COVID-19.

## Methods

### Data

We obtained country-level broad-spectrum oral antibiotic sales data from the IQVIA MIDAS® database.^33^ MIDAS data are collected on a monthly basis from a sample of pharmacies and other outlets through which antibiotics are dispensed and aggregated to project the total volume of monthly antibiotic sales in each country. We used monthly MIDAS data on sales volumes of four broad-spectrum antibiotics: cephalosporins (WHO Anatomical Therapeutic Chemical or ATC code J01D1), penicillins (J01C1), macrolides (J01F0), and tetracyclines (J01A0). Sales were expressed in standard units that represent a single dose, including a pill, capsule, or unit of liquid. Data were available for 71 countries of the world, and a continent-wise list of these countries are provided in the Supplementary appendix. We examined global trends in antibiotic sales from January 2018 to May 2022. Our regression analysis included data from the pandemic period of March 2020–May 2022.

We combined the antibiotic sales data with COVID-19 data obtained from Our World in Data (OWD).^34^ The OWD database provides COVID-19 statistics (number of reported COVID-19 cases and deaths) for each country, which are drawn from the Johns Hopkins University COVID-19 database.^35^^,^^36^ OWD compiles additional indicators such as COVID-19 hospitalizations, intensive care unit admissions, and COVID-19 vaccinations from various sources including government reports. Finally, OWD compiles demographic, economic, healthcare access, and health outcome indicators such as population size, median age, per capita gross domestic product, per capita availability of hospital beds, and prevalence of smoking for each country from international and local agencies, including the World Bank and United Nations. Data on COVID-19 indicators were available daily, while the background data for countries were either available as point estimates (most recent available) or converted into a time-invariant mean value.

Using population data from OWD, we converted the monthly aggregate antibiotic sales volume data from MIDAS into monthly sales per 1000 people. We considered two COVID-19 indicators from OWD: reported daily number of new cases and new vaccinations. To match the level and frequency of the MIDAS data, we aggregated these indicators into monthly data expressed per 1000 people. Sales and COVID-19 indicators were then converted into a natural logarithm for easy interpretation in regression analysis. COVID-19 cases data were available for the full duration between March 2020 and May 2022, while vaccination data were available for December 2020–May 2022. All data used in this study were anonymous and aggregated at the country level, and no ethical approval and informed consent were necessary.

### Statistical analysis

We graphically examined the global trends in the sales of each antibiotic (averaged across all countries) during January 2018 through May 2022 along with trends in COVID-19 cases starting from January 2020. We then conducted pooled and panel data fixed-effects regressions of log monthly antibiotic sales per 1000 people on monthly COVID-19 cases per 1000 people. We started with an ordinary least squares model of the following form (commonly known as a pooled regression in panel data analysis)^37^:

Equation (1) $$ABX_{mc}=\alpha_{1}+\beta_{1}{Cases}_{mc}+\gamma_{1}X_{c}+u_{mc}$$

where $ABX_{mc}$ denotes log sales per 1000 people of an antibiotic during the *m-*th month in the *c-*th country. ${Cases}_{mc}$ denotes log COVID-19 cases per 1000 people during the *m-*th month in the *c-*th country. $X_{c}$ denotes a set of country-level covariates that includes log of population, population density (per square km), median age of population (years), gross domestic product per capita (constant 2011 international dollars, purchasing power parity), percentage of population of age 70 years or older, cardiovascular death rate in 2017 (per 100,000 people), diabetes prevalence rate in 2017 (age 20–79 years), hospital beds per 1000 people, percentage of male and female smokers, life expectancy at birth in 2019, human development index value in 2019, and COVID-19 stringency index. The stringency index is a composite measure of government response to COVID-19, including closures and travel bans, on a scale of 0–100 (higher value implies stricter measures). Binary indicators of continent (Asia, Europe, North America, Oceania, and South America) were also included in *X*. The coefficient $\beta_{1}$ represents the estimated association between COVID-19 cases and antibiotics sales. $\alpha_{1}$ represents the intercept term, while $\gamma_{1}$ is a vector of regression coefficients representing associations of the covariates in *X.* The normally distributed error term of the model is denoted by $u_{mc}$.

We separately estimated another pooled regression model by accounting for COVID-19 vaccinations as follows:

Equation (2) $$ABX_{mc}=\alpha_{2}+\beta_{2}{Cases}_{mc}+\gamma_{2}{Vaccines_{mc}+\delta_{2}X}_{c}+\mu_{mc}$$

where $Vaccines_{mc}$ denotes log COVID-19 vaccinations per 1000 people during the *m-*th month in the *c-*th country. The coefficients $\beta_{2}$ and $\gamma_{2}$ represent estimated associations of antibiotic sales with COVID-19 cases and vaccinations respectively.

We also estimated a set of country fixed-effects regression models (without COVID-19 vaccinations), described as follows:

Equation (3) $$ABX_{mc}=\alpha_{3}+\beta_{3}{Cases}_{mc}{+\gamma_{3}X}_{c}+\delta_{3}FE_{c}+\nu_{mc}$$

where $FE_{c}$ is a set of country fixed effects (equivalent to binary indicators of countries). Note that the covariate set *X* was subsumed (removed from the model) by the fixed effects as the latter accounted for the former along with all other unobserved country-specific factors. The other parameters of the model are akin to those in Equation (1).

Finally, we estimated a set of country fixed-effects regression models, which also included COVID-19 vaccinations as follows:

Equation (4) $$ABX_{mc}=\alpha_{4}+\beta_{4}{Cases}_{mc}+\gamma_{4}{Vaccines_{mc}+\delta_{4}X}_{c}+\theta_{4}FE_{c}+\tau_{mc}$$

The parameters of the model represent associations similar to those in Equation (3). We estimated each regression model separately for the four broad-spectrum oral antibiotics and a fifth model with all four antibiotics (combined sales volume). Analysis was done at the global level for all countries and separately for each continent for the periods of March 2020–May 2022 for models (1) and (3) and December 2020–May 2022 for models (2) and (4). Standard errors of all regression models were clustered at the country level, and we considered p < 0.05 for statistical significance. STATA 16.1 was used for analysis.

### Role of the funding source

The funder had no role in study design, analysis, preparation of the manuscript, or the decision to submit for publication. SP and AN had access to the data, and all authors accepted responsibility to submit for publication.

## Results

**Fig. 1:**
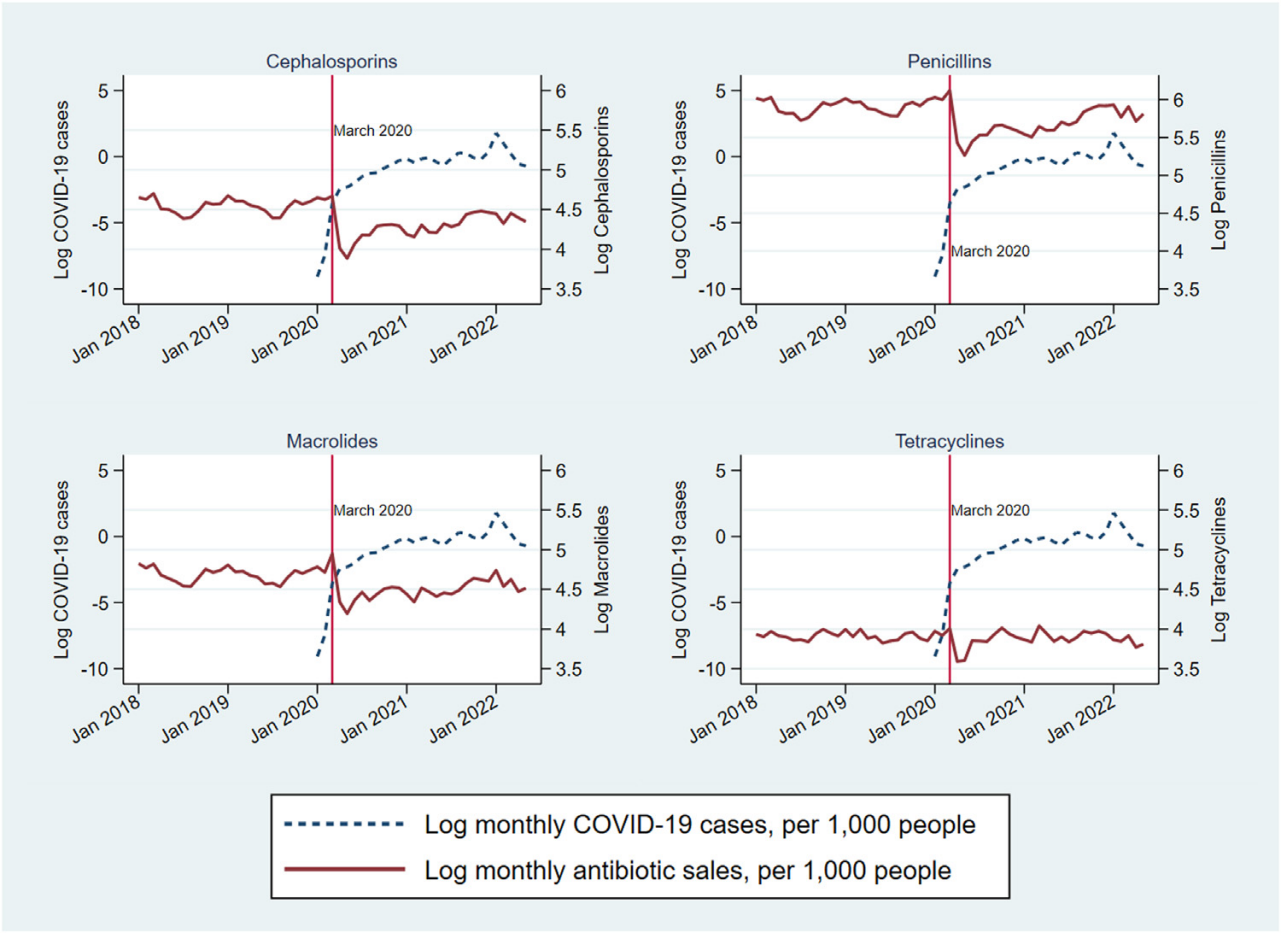
**COVID-19 cases and oral broad-spectrum antibiotic use in 71 countries, January 2018–May 2022**. Note: Data are monthly global (71 countries) averages, obtained from IQVIA MIDAS and Our World in Data.

Fig. 1 presents trends in monthly antibiotic sales and COVID-19 cases per 1000 people averaged across all countries. Sales of all four antibiotics fell substantially in April and May 2020 as compared with the earlier period of January 2018–March 2020. Sales started to increase again in June 2020, gradually returning to pre-pandemic levels.

Table 1 presents pooled (model 1) and fixed-effects (model 3) regression results without accounting for vaccinations, while Table 2 presents those including vaccinations (models 2 and 4). In the pooled model without vaccinations (regression model 1), a 10% increase in monthly COVID-19 cases was associated with 0.52% (95% confidence interval: 0.01, 1.02; p = 0.045) higher monthly penicillin sales and 0.44% (0.06, 0.82; p = 0.023) higher monthly macrolide sales per 1000 people. In fixed-effects regression models (model 3), a 10% increase in monthly COVID-19 cases was associated with 0.27% (0.13, 0.41; p = 0.0002), 0.33% (0.23, 0.43; p < 0.0001), 0.45% (0.3, 0.59; p < 0.0001), and 0.32% (0.21, 0.43; p < 0.0001) higher sales of cephalosporins, penicillins, macrolides, and all four antibiotics per 1000 people respectively. No significant associations were seen for tetracyclines in either model and for macrolides and all four antibiotics in pooled regression (model 1).

**Table 1:** Regression analysis of COVID-19 cases and oral broad-spectrum antibiotic use, March 2020–May 2022.

	Cephalosporins	Penicillins	Macrolides	Tetracyclines	All four antibiotics
*Pooled regression (model 1):*					
Log COVID-19 cases	0.032 (0.032)	0.052 (0.025)∗	0.044 (0.019)∗	0.002 (0.023)	0.036 (0.022)
Log of population	0.202 (0.07)∗∗	0.105 (0.055)	0.168 (0.038)∗∗∗∗	0.16 (0.077)∗	0.144 (0.048)∗∗
Population density (per square km)	−0.002 (0.001)	0.001 (0.001)	0.0003 (0.0005)	0.001 (0.001)	0.0003 (0.0006)
Median age of population	0.038 (0.051)	0.041 (0.043)	0.091 (0.027)∗∗∗	0.042 (0.056)	0.041 (0.034)
Gross domestic product per capita (constant 2011 international dollars)	0.000016 (0.0000)	0.000006 (0.0000)	0.0000148 (0.0000)∗	0.000015 (0.0000)	0.0000092 (0.0000)
% of population of age ≥70 years	−0.035 (0.074)	−0.065 (0.056)	−0.103 (0.035)∗∗	−0.015 (0.067)	−0.04 (0.046)
Cardiovascular death rate per 100,000 people	0.002 (0.002)	−0.00022 (0.0014)	0.002 (0.001)∗	−0.001 (0.002)	0.001 (0.001)
Diabetes prevalence rate (age 20–79 years)	0.007 (0.045)	0.01 (0.037)	−0.029 (0.029)	−0.071 (0.053)	−0.012 (0.033)
Hospital beds per 1000 people	0.173 (0.081)∗	−0.011 (0.042)	0.049 (0.028)	−0.092 (0.052)	0.002 (0.032)
% of smokers, female	0.038 (0.017)∗	0.007 (0.011)	0.023 (0.01)∗	0.004 (0.016)	0.009 (0.009)
% of smokers, male	−0.008 (0.013)	0.018 (0.009)∗	−0.009 (0.007)	0.016 (0.01)	0.008 (0.008)
Life expectancy at birth	0.15 (0.059)∗	0.049 (0.041)	0.083 (0.031)∗∗	−0.008 (0.044)	0.064 (0.038)
Human development index	−11.781 (3.883)∗∗	−4.703 (2.478)	−6.28 (2.41)∗∗	−1.532 (4.857)	−5.108 (2.394)∗
COVID-19 stringency index	0.002 (0.023)	−0.022 (0.017)	−0.016 (0.011)	−0.061 (0.02)∗∗	−0.027 (0.014)
Asia	0.608 (0.519)	−0.894 (0.33)∗∗	−0.915 (0.33)∗∗	−0.153 (0.465)	−0.536 (0.313)
Europe	−1.138 (0.708)	−0.703 (0.478)	−1.316 (0.372)∗∗∗	−0.665 (0.562)	−0.86 (0.417)∗
North America	−0.398 (0.606)	−1.033 (0.453)∗	−1.384 (0.335)∗∗∗∗	−0.063 (0.6)	−0.967 (0.425)∗
Oceania	1.261 (0.811)	0.439 (0.526)	−0.331 (0.34)	0.934 (0.521)	0.406 (0.426)
South America	−0.106 (0.608)	−0.217 (0.401)	−0.969 (0.315)∗∗	−0.252 (0.432)	−0.397 (0.355)
Constant term	−3.533 (3.595)	3.86 (2.733)	−1.206 (1.97)	5.014 (3.61)	3.234 (2.539)
*Fixed-effects panel regression (model 3):*					
Log COVID-19 cases	0.027 (0.007)∗∗∗	0.033 (0.005)∗∗∗∗	0.045 (0.007)∗∗∗∗	0.013 (0.007)	0.032 (0.005)∗∗∗∗
Constant term	4.284 (0.003)	5.697 (0.002)	4.512 (0.003)	3.931 (0.003)	6.353 (0.002)
Sample size	1662	1663	1663	1663	1663

Note: Data are from IQVIA MIDAS and Our World in Data. The outcome variable of the regression is log monthly antibiotic sales per 1000 people. Log COVID-19 cases are per 1000 people, monthly. Standard errors are in parentheses. ∗p < 0.05, ∗∗p < 0.01, ∗∗∗p < 0.001, ∗∗∗∗p < 0.0001.

**Table 2:** Regression analysis of COVID-19 cases, vaccinations, and oral broad-spectrum antibiotic use, December 2020–May 2022.

	Cephalosporins	Penicillins	Macrolides	Tetracyclines	All four antibiotics
*Pooled regression (model 2):*					
Log COVID-19 cases	0.061 (0.043)	0.07 (0.035)	0.076 (0.022)∗∗	0.018 (0.032)	0.057 (0.03)
Log COVID-19 vaccinations	−0.015 (0.016)	−0.013 (0.011)	−0.021 (0.009)∗	−0.004 (0.013)	−0.013 (0.009)
Log of population	0.206 (0.071)∗∗	0.117 (0.055)∗	0.173 (0.037)∗∗∗∗	0.167 (0.074)∗	0.151 (0.049)∗∗
Population density (per square km)	−0.003 (0.001)	0.00043 (0.0008)	0.000223 (0.0005)	0.001 (0.001)	0.00014 (0.0006)
Median age of population	0.045 (0.052)	0.037 (0.044)	0.092 (0.027)∗∗∗	0.037 (0.055)	0.041 (0.034)
Gross domestic product per capita (constant 2011 international dollars)	0.0000183 (0.0000)	0.0000078 (0.0000)	0.000015 (0.0000)∗∗	0.000015 (0.0000)	0.00001 (0.0000)
% of population of age ≥70 years	−0.042 (0.075)	−0.063 (0.057)	−0.1 (0.036)∗∗	−0.001 (0.067)	−0.04 (0.047)
Cardiovascular death rate per 100,000 people	0.003 (0.002)	0 (0.001)	0.002 (0.001)∗	−0.001 (0.002)	0.001 (0.001)
Diabetes prevalence rate (age 20–79 years)	0.003 (0.043)	0.006 (0.037)	−0.03 (0.027)	−0.076 (0.05)	−0.016 (0.032)
Hospital beds per 1000 people	0.178 (0.084)∗	−0.006 (0.042)	0.051 (0.027)	−0.103 (0.05)∗	0.003 (0.032)
% of smokers, female	0.04 (0.017)∗	0.006 (0.012)	0.024 (0.01)∗	0.006 (0.015)	0.01 (0.009)
% of smokers, male	−0.009 (0.013)	0.016 (0.009)	−0.01 (0.007)	0.014 (0.01)	0.006 (0.008)
Life expectancy at birth	0.147 (0.059)∗	0.048 (0.042)	0.076 (0.03)∗	−0.019 (0.044)	0.06 (0.038)
Human development index	−12.414 (3.973)∗∗	−4.514 (2.462)	−6.221 (2.239)∗∗	−0.745 (4.538)	−4.956 (2.282)∗
COVID-19 stringency index	0.005 (0.023)	−0.018 (0.016)	−0.01 (0.011)	−0.058 (0.019)∗∗	−0.023 (0.013)
Asia	0.52 (0.519)	−0.994 (0.345)∗∗	−0.93 (0.323)∗∗	−0.083 (0.442)	−0.596 (0.315)
Europe	−1.256 (0.714)	−0.794 (0.49)	−1.355 (0.36)∗∗∗	−0.802 (0.547)	−0.958 (0.422)∗
North America	−0.473 (0.613)	−1.144 (0.471)∗	−1.412 (0.321)∗∗∗∗	−0.158 (0.607)	−1.063 (0.435)∗
Oceania	1.234 (0.819)	0.315 (0.55)	−0.317 (0.346)	0.832 (0.508)	0.305 (0.438)
South America	−0.259 (0.618)	−0.319 (0.422)	−0.993 (0.309)∗∗	−0.369 (0.424)	−0.493 (0.367)
Constant term	−3.064 (3.556)	3.681 (2.747)	−1.133 (1.902)	5.164 (3.709)	3.282 (2.569)
*Fixed-effects panel regression (model 4):*					
Log COVID-19 cases	0.016 (0.007)∗	0.023 (0.007)∗∗	0.06 (0.012)∗∗∗∗	0.006 (0.008)	0.029 (0.007)∗∗∗
Log COVID-19 vaccinations	0.004 (0.006)	0.01 (0.005)∗	−0.005 (0.006)	0.006 (0.007)	0.005 (0.004)
Constant term	4.286 (0.023)	5.691 (0.02)	4.506 (0.026)	3.911 (0.03)	6.346 (0.018)
Sample size	1054	1055	1055	1055	1054

Note: Data are from IQVIA MIDAS and Our World in Data. The outcome variable of the regression is log monthly antibiotic sales per 1000 people. Log COVID-19 cases and vaccinations are per 1000 people, monthly. Standard errors are in parentheses. ∗p < 0.05, ∗∗p < 0.01, ∗∗∗p < 0.001, ∗∗∗∗p < 0.0001.

In the pooled regression including COVID-19 vaccinations (model 2), a 10% increase in monthly COVID-19 cases was associated with 0.76% (0.32, 1.19; p = 0.0012) higher macrolide sales, while a 10% increase in monthly COVID-19 vaccinations was associated with 0.21% (−0.38, −0.03; p = 0.022) lower macrolide sales per 1000 people. No significant associations were evident for other antibiotics. In fixed-effects regression including COVID-19 vaccinations (model 4), a 10% increase in monthly COVID-19 cases was associated with 0.16% (0.02, 0.29; p = 0.0248), 0.23% (0.09, 0.38; p = 0.0025), 0.6% (0.35, 0.84; p < 0.0001), and 0.29% (0.15, 0.43; p = 0.0001) higher sales of cephalosporins, penicillins, macrolides, and all four antibiotics combined per 1000 people respectively. For penicillins, a 10% increase in monthly COVID-19 vaccinations was associated with 0.1% (0.01, 0.2; p = 0.0316) higher sales per 1000 people. No other significant associations were observed.

In continent-specific fixed-effects regression analysis (Table 3, Table 4), the largest significant associations were for macrolides, with 0.39% (0.04, 0.73; p = 0.0466), 0.78% (0.53, 1.03; p < 0.0001), 1.33% (0.63, 2.04; p = 0.0139), and 1.48% (1.1, 1.86; p = 0.0016) increases in sales for a 10% increase in COVID-19 cases in Asia, Europe, North America, and Africa respectively (accounting for vaccination). Across all regression models by continent, significant associations between antibiotics sales and COVID-19 cases were primarily seen in European countries, and among the four antibiotics, significant associations with COVID-19 cases were mainly observed for macrolides. Vaccinations had statistically significant associations with antibiotic sales in some cases, but no clear pattern emerged.

**Table 3:** Regression analysis (without accounting for COVID-19 vaccines) of COVID-19 cases and oral broad-spectrum antibiotic use by region, March 2020–May 2022.

	Cephalosporins	Penicillins	Macrolides	Tetracyclines	All four antibiotics
*Pooled regression, by region (model 1):*					
Africa	0.045 (0.022)∗	0.013 (0.022)	0.089 (0.019)∗∗∗∗	0.023 (0.019)	0.03 (0.02)
Asia	−0.013 (0.015)	0.005 (0.012)	0.009 (0.014)	−0.008 (0.014)	0.006 (0.014)
Europe	0.042 (0.019)∗	0.066 (0.011)∗∗∗∗	0.062 (0.007)∗∗∗∗	0.013 (0.009)	0.056 (0.008)∗∗∗∗
North America	−0.005 (0.017)	0.029 (0.021)	0.042 (0.025)	0.013 (0.013)	0.023 (0.018)
Oceania	0.009 (0.006)	0.005 (0.012)	−0.006 (0.007)	0.005 (0.007)	0.003 (0.008)
South America	0.00041 (0.009)	−0.002 (0.015)	0.059 (0.019)∗∗	0.004 (0.012)	0.008 (0.012)
*Fixed-effects panel regression, by region (model 3):*					
Africa	0.045 (0.021)	0.013 (0.011)	0.089 (0.019)∗∗	0.023 (0.016)	0.03 (0.01)∗
Asia	0.003 (0.01)	0.018 (0.008)∗	0.023 (0.01)∗	0.006 (0.017)	0.015 (0.009)
Europe	0.054 (0.011)∗∗∗∗	0.058 (0.006)∗∗∗∗	0.063 (0.009)∗∗∗∗	0.021 (0.009)∗	0.056 (0.006)∗∗∗∗
North America	−0.005 (0.014)	0.029 (0.011)∗	0.042 (0.014)∗	0.013 (0.005)	0.023 (0.01)
Oceania	0.009 (0.002)	0.005 (0.016)	−0.006 (0.007)	0.005 (0.001)	0.003 (0.01)
South America	0.00041 (0.014)	−0.002 (0.017)	0.059 (0.023)	0.004 (0.009)	0.008 (0.012)

Note: Data are from IQVIA MIDAS and Our World in Data. The outcome variable of the regression is log monthly antibiotic sales per 1000 people. Analysis was done separately for countries in each region. Only the estimated regression coefficient of COVID-19 cases per 1000 is shown to preserve space. Standard errors are in parentheses. ∗p < 0.05, ∗∗p < 0.01, ∗∗∗p < 0.001, ∗∗∗∗p < 0.0001.

**Table 4:** Regression analysis of COVID-19 cases, vaccinations, and oral broad-spectrum antibiotic use by region, December 2020–May 2022.

	Cephalosporins	Penicillins	Macrolides	Tetracyclines	All four antibiotics
*Pooled regression, by region (model 2):*					
Africa: COVID-19 cases	0.035 (0.02)	0.02 (0.024)	0.148 (0.02)∗∗∗∗	−0.008 (0.022)	0.042 (0.02)∗
Asia: COVID-19 cases	−0.003 (0.025)	0.007 (0.021)	0.031 (0.023)	−0.003 (0.024)	0.014 (0.022)
Europe: COVID-19 cases	0.036 (0.031)	0.059 (0.016)∗∗∗	0.079 (0.008)∗∗∗∗	0.01 (0.013)	0.056 (0.011)∗∗∗∗
North America: COVID-19 cases	−0.007 (0.025)	0.076 (0.027)∗∗	0.133 (0.025)∗∗∗∗	−0.007 (0.019)	0.065 (0.022)∗∗
Oceania: COVID-19 cases	0 (0.004)	−0.016 (0.013)	−0.018 (0.008)∗	−0.003 (0.006)	−0.011 (0.009)
South America: COVID-19 cases	−0.01 (0.011)	−0.021 (0.021)	0.1 (0.026)∗∗∗	−0.044 (0.013)∗∗	−0.002 (0.017)
Africa: COVID-19 vaccinations	0.041 (0.013)∗∗	0.031 (0.014)∗	0 (0.013)	−0.049 (0.019)∗	0.025 (0.013)∗
Asia: COVID-19 vaccinations	0.007 (0.02)	0.019 (0.017)	0.001 (0.019)	0.032 (0.029)	0.012 (0.018)
Europe: COVID-19 vaccinations	0.016 (0.022)	−0.004 (0.014)	−0.011 (0.008)	0.002 (0.011)	−0.003 (0.009)
North America: COVID-19 vaccinations	0.012 (0.012)	0.016 (0.013)	−0.024 (0.018)	0.009 (0.012)	0.004 (0.011)
Oceania: COVID-19 vaccinations	−0.009 (0.013)	0.012 (0.034)	0.014 (0.023)	0.014 (0.016)	0.01 (0.024)
South America: COVID-19 vaccinations	0.009 (0.006)	0.034 (0.01)∗∗	0.041 (0.014)∗∗	0.031 (0.017)	0.028 (0.008)∗∗∗
*Fixed-effects panel regression, by region (model 4):*					
Africa: COVID-19 cases	0.035 (0.026)	0.02 (0.026)	0.148 (0.019)∗∗	−0.008 (0.013)	0.042 (0.022)
Asia: COVID-19 cases	0.005 (0.013)	0.014 (0.015)	0.039 (0.018)∗	0.005 (0.019)	0.016 (0.013)
Europe: COVID-19 cases	0.033 (0.013)∗	0.046 (0.01)∗∗∗∗	0.078 (0.013)∗∗∗∗	0.018 (0.013)	0.052 (0.008)∗∗∗∗
North America: COVID-19 cases	−0.007 (0.03)	0.076 (0.034)	0.133 (0.036)∗	−0.007 (0.022)	0.065 (0.031)
Oceania: COVID-19 cases	0 (0.002)	−0.016 (0.008)	−0.018 (0.005)	−0.003 (0)	−0.011 (0.007)
South America: COVID-19 cases	−0.01 (0.016)	−0.021 (0.022)	0.1 (0.052)	−0.044 (0.024)	−0.002 (0.02)
Africa: COVID-19 vaccinations	0.041 (0.023)	0.031 (0.013)	0 (0.007)	−0.049 (0.019)	0.025 (0.011)
Asia: COVID-19 vaccinations	0.004 (0.013)	0.016 (0.014)	−0.002 (0.015)	0.028 (0.022)	0.011 (0.013)
Europe: COVID-19 vaccinations	−0.001 (0.008)	0.003 (0.006)	−0.011 (0.006)	0.001 (0.004)	−0.001 (0.005)
North America: COVID-19 vaccinations	0.012 (0.013)	0.016 (0.008)	−0.024 (0.021)	0.009 (0.017)	0.004 (0.006)
Oceania: COVID-19 vaccinations	−0.009 (0.005)	0.012 (0.003)	0.014 (0.001)∗	0.014 (0.011)	0.01 (0)∗
South America: COVID-19 vaccinations	0.009 (0.007)	0.034 (0.014)	0.041 (0.024)	0.031 (0.022)	0.028 (0.011)

Note: Data are from IQVIA MIDAS and Our World in Data. The outcome variable of the regression is log monthly antibiotic sales per 1000 people. Analysis was done separately for countries in each region. Only the estimated regression coefficients of COVID-19 cases and vaccinations per 1000 are shown. Standard errors are in parentheses. ∗p < 0.05, ∗∗p < 0.01, ∗∗∗p < 0.001, ∗∗∗∗p < 0.0001.

## Discussion

Antibiotic overuse continues to be a major global health challenge. We used pharmaceutical sales data from 71 countries and employed panel data regression models to examine the associations of antibiotic sales during 2020–2022 with COVID-19 cases and vaccinations. A large reduction in per capita consumption of all four antibiotics studied occurred during the first few months of the pandemic (Fig. 1) as compared with 2018 levels. Antibiotic consumption is known to be seasonal, with increased use during the winter months and a marked reduction starting in later spring and early summer every year.^38^ However, the reductions in antibiotics sales during the first months of the pandemic were larger than seasonal reductions seen in previous years, and were likely due to NPIs for COVID-19 control such as lockdowns, closures, and restrictions on travel and gatherings.

In fixed-effects regression analysis, which accounted for time-invariant observed and unobserved country characteristics, sales of cephalosporins, penicillins, macrolides, and all four antibiotics combined were consistently associated positively with COVID-19 cases. No associations were seen for tetracyclines. COVID-19 vaccinations were inversely associated with macrolide sales in the pooled analysis but positively associated with penicillin sales in the fixed-effects models. The magnitude of the estimated associations was smaller for vaccinations than for cases.

There are important mediating factors for the estimated associations between COVID-19 cases and antibiotic use. Systematic review and meta-analysis studies have documented rampant antibiotic overuse in patients with COVID-19 globally in both hospital and community settings.^11^^,^^15^^,^^16^ A review of national treatment guidelines found that even in mid-2021, many African countries were recommending antibiotics to manage COVID-19 cases.^39^ A systematic review estimated prevalence rates of self-medication with antibiotics and other drugs to prevent or manage COVID-19 to be as high as 88% in some LMICs,^24^ while another study from India attributed 216 million excess doses of antibiotics sold, including 38 million excess doses of azithromycin, to the pandemic in 2020.^25^

The effect of the pandemic on routine childhood immunization delivery may have additional implications for antibiotic use. Globally, the number of third doses of diphtheria, pertussis, and tetanus vaccine administered to children during the first half of 2020 was 33% lower than during the first half of 2019.^29^ In India, coverage rates of individual vaccine doses were 2%–10% lower with 3%–5% longer delays in timely vaccine receipt during the first year of the pandemic than in 2019.^32^ Children who missed vaccine doses remained at an elevated risk of contracting vaccine-preventable diseases, potentially increasing future antibiotic use.^40^

While antibiotic use in patients with COVID-19 was high, the prevalence of other diseases and economic factors may also have affected the overall sales of antibiotics in each country. Antibiotic prescribing was 13%–56% lower in the United States and 27% lower in Canada through the end of 2020 than in other years.^41^, ^42^, ^43^ Due to NPIs for COVID-19 control such as masking, lockdowns, school closures, and restrictions on travel and gatherings, virtually no annual influenza season occurred in 2020–2021 and only a mild season occurred in 2021–2022.^18^, ^19^, ^20^, ^21^ Influenza-like illnesses are a major driver of antibiotic use globally during winter months, which includes both appropriate use for secondary bacterial infections and inappropriate use.^44^ Finally, antibiotic use is positively associated with standard of living across countries,^9^ and the economic hardship imposed globally by the pandemic^45^ may have reduced access to and the sales of antibiotics.

Governments around the world have eased COVID-related NPIs in between COVID-19 surges and more so since the introduction of COVID-19 vaccines at the end of 2020. As a result, normalcy has been returning slowly, bringing with it surges of non-COVID infections. One such example is the respiratory syncytial virus (RSV), which resurged strongly in 2021, possibly contributing to increased antibiotic use.^26^, ^27^, ^28^ Higher incidence of RSV and other diseases may be the driving factor for the estimated positive association between COVID-19 vaccinations and penicillin sales in our fixed-effects regression model. A 2021 study from Israel found that a third of paediatric patients with RSV received unnecessary antibiotics, and penicillins were the most commonly prescribed antibiotics (39% of prescriptions).^46^

In additional analysis, we examined if COVID-19 vaccinations might be associated with antibiotic use with a time lag. At the individual level, immunity conferred by a COVID-19 vaccine takes up to two weeks to fully develop, and secondary immunity from widespread vaccinations at the community level can take much longer. We repeated our analysis by including lagged values of monthly COVID-19 vaccinations per 1000 people (1, 2, and 3 months of lag) among the regression covariates. The estimated coefficients of the lagged indicators were not statistically significant.

Our study has important policy implications. Experts have raised the alarm for antibiotic overuse and misuse for COVID-19 treatment and their longer-term implications for global antibiotic resistance.^11^^,^^39^^,^^47^, ^48^, ^49^, ^50^ Our findings show that the overall effect of the pandemic on increasing private broad-spectrum antibiotic use so far has been modest, possibly due to fewer non-COVID infections. However, preventing unnecessary treatment of COVID-19 cases with antibiotics remains essential. COVID-19 will likely become endemic eventually and similarly virulent as the common cold,^51^ and medical guidelines and government policies must stop it from becoming another influenza-like illness for which antibiotics are continually and inappropriately prescribed.^44^

Our analysis has some limitations. IQVIA data were available only for 71 countries and not all continents were represented equally. For example, South Africa was the only country with data from sub-Saharan Africa. Also, some large countries such as Bangladesh and Myanmar were not included. Second, we incorporated a series of country-level determinants and fixed effects of monthly antibiotic sales. However, other time-varying factors that can potentially affect our outcome indicators, such as trends in non-COVID infections, could not be incorporated due to lack of data. In addition, reported COVID-19 case data are known to suffer from undercounting due to incorrect reporting and the use of home-based COVID test kits.^52^ A recent study estimated that 60% of COVID-19 cases may have gone unreported in the United States,^53^ while another global study estimated that in many large countries reported cases may represent only 1%–2% of the actual number of infections.^54^ Finally, our study does not capture the potential longer-term associations between COVID-19 and antibiotic use beyond May 2022. Research using long-term data are important for antibiotic stewardship and resistance control.

## Contributors

AN, SP, and DB designed the study. SP obtained the data, and AN analysed the data and wrote the first version of the manuscript. All authors critically evaluated and edited the manuscript. SP and AN had access to and have verified the data. All authors approved the final version for submission and were responsible for the decision to submit.

## Data sharing statement

Data are available from IQVIA, and can be obtained by contacting them through https://www.iqvia.com/solutions/commercialization/brand-strategy-and-management/market-measurement/midas.

## Declaration of interests

SP was an employee of the GlaxoSmithKline group of companies when this research was conducted, and is currently an employee of Moderna Inc. DB has previously received research support or personal fees from 10.13039/100004330GlaxoSmithKline plc, 10.13039/100004334Merck, 10.13039/100004319Pfizer, and Sanofi-Pasteur related to value-of-vaccination research, but not for this study. AN received travel support from 10.13039/100004330GlaxoSmithKline plc for attending a conference related to the work presented in this manuscript.
