# Supplementary material for: Global antibiotic use during the COVID-19 pandemic: analysis of pharmaceutical sales data from 71 countries, 2020–2022
Source: eClinicalMedicine. 2023 Feb 6;57:101848. doi: 10.1016/j.eclinm.2023.101848 (PMC9900305; doi:10.1016/j.eclinm.2023.101848)
Supplement: Supplementary Appendix [file mmc1.docx]

**Supplementary Appendix**

Table A1: Continent-wise list of countries with available IQVIA data

| **Continent** | **Country** |
| --- | --- |
| Africa | Algeria |
| Africa | Egypt |
| Africa | Morocco |
| Africa | South Africa |
| Africa | Tunisia |
| Asia | China |
| Asia | Hong Kong |
| Asia | India |
| Asia | Indonesia |
| Asia | Japan |
| Asia | Jordan |
| Asia | Korea |
| Asia | Kuwait |
| Asia | Lebanon |
| Asia | Malaysia |
| Asia | Pakistan |
| Asia | Philippines |
| Asia | Saudi Arabia |
| Asia | Taiwan |
| Asia | Thailand |
| Asia | Turkey |
| Asia | UAE |
| Asia | Vietnam |
| Europe | Austria |
| Europe | Belarus |
| Europe | Belgium |
| Europe | Bulgaria |
| Europe | Croatia |
| Europe | Czech Republic |
| Europe | Denmark |
| Europe | Finland |
| Europe | France |
| Europe | Germany |
| Europe | Greece |
| Europe | Hungary |
| Europe | Ireland |
| Europe | Italy |
| Europe | Latvia |
| Europe | Luxembourg |
| Europe | Netherlands |
| Europe | Norway |
| Europe | Poland |
| Europe | Portugal |
| Europe | Romania |
| Europe | Russia |
| Europe | Serbia |
| Europe | Slovakia |
| Europe | Slovenia |
| Europe | Spain |
| Europe | Sweden |
| Europe | Switzerland |
| Europe | UK |
| North America | Canada |
| North America | Costa Rica |
| North America | El Salvador |
| North America | Guatemala |
| North America | Honduras |
| North America | Mexico |
| North America | Nicaragua |
| North America | Panama |
| North America | Puerto Rico |
| North America | USA |
| Oceania | Australia |
| Oceania | New Zealand |
| South America | Argentina |
| South America | Brazil |
| South America | Chile |
| South America | Colombia |
| South America | Ecuador |
| South America | Peru |
| South America | Uruguay |
